# Spatiotemporal Distribution and Meteorological Determinants of Lumpy Skin Disease (LSD) Occurrence in Bangladesh From 2020 to 2023

**DOI:** 10.1155/tbed/4259023

**Published:** 2025-11-14

**Authors:** Md Jisan Ahmed, Kazi Estieque Alam, Faisol Talukdar, Md Ismile Hossain Bhuiyan, Prajwal Bhandari, Ritu Chalise, Dodul Mahamud, Md Imran Hossain, Mirza Synthia Sabrin, Md. Tazul Islam Chowdhury, Md. Jahangir Alam, Delower Hossain

**Affiliations:** ^1^Association of Coding, Technology, and Genomics (ACTG), Sher-e-Bangla Agricultural University (SAU), Dhaka 1207, Bangladesh; ^2^Department of Pathology, Faculty of Animal Science and Veterinary Medicine, Sher-e-Bangla Agricultural University (SAU), Dhaka 1207, Bangladesh; ^3^Department of Livestock Services (DLS), Government of Bangladesh, Dhaka 1215, Bangladesh; ^4^Department of Animal Production and Management, Faculty of Animal Science and Veterinary Medicine, Sher-e-Bangla Agricultural University (SAU), Dhaka 1207, Bangladesh; ^5^Department of Microbiology and Parasitology, Faculty of Animal Science and Veterinary Medicine, Sher-e-Bangla Agricultural University (SAU), Dhaka 1207, Bangladesh; ^6^Department of Agricultural Chemistry, Faculty of Agriculture, Sher-e-Bangla Agricultural University (SAU), Dhaka 1207, Bangladesh; ^7^Department of Medicine and Public Health, Faculty of Animal Science and Veterinary Medicine, Sher-e-Bangla Agricultural University (SAU), Dhaka 1207, Bangladesh

**Keywords:** Bangladesh, capripox virus, cattle, climate, lumpy skin disease, spatiotemporal

## Abstract

Lumpy skin disease (LSD) is a transboundary viral disease affecting cattle and buffaloes caused by LSD virus (LSDV), which belongs to the *Capripoxvirus* genus. The disease was first detected in Bangladesh in 2019 and has since become endemic. This study focuses on analyzing the spatiotemporal distribution of LSD cases and examining the relationships between LSD incidence and climatic variables using data collected from 2020 to 2023 in Bangladesh. LSD incidence data from the Department of Livestock Services (DLS) and climate data from the Bangladesh Meteorological Department (BMD) were analyzed via descriptive statistics, box plots, and spatial methods. Hotspots were identified via Getis-Ord Gi^*∗*^ statistics (Gi^*∗*^), whereas Global Moran's *I* and local indicators of spatial association (LISA) detected spatial autocorrelation. Associations with climate variables were assessed using Spearman's correlation and modeled through Poisson, quasi-Poisson, and negative binomial regressions. LSD cases in Bangladesh exhibited substantial temporal and spatial variability between 2020 and 2023, with the highest peaks occurring in 2023, mainly from May to November. Spatial analyses revealed evolving hotspots shifting from southeastern coastal districts (e.g., Chattogram, Cox's Bazar) to northern and northwestern regions (e.g., Rajshahi, Rangpur) over time. Spearman's rank correlation revealed significant positive associations between LSD cases and rainfall (*r* = 0.29), relative humidity (*r* = 0.37), minimum temperature (*r* = 0.29), and wind speed (*r* = 0.22). Regression analyses revealed consistent meteorological influences. In the Poisson model, average sunshine (incidence rate ratio [IRR] = 3.57), minimum temperature (IRR = 1.687), and wind speed (IRR = 2.639) significantly increased LSD cases, whereas average temperature had a strong protective effect (IRR = 0.362). This study reveals strong seasonal and climatic influences on LSD outbreaks in Bangladesh, with peaks occurring during the monsoon season and shifting hotspots from southeast to north. These findings emphasize the importance of climate-based surveillance and targeted control measures.

## 1. Introduction

In recent years, the increasing incidence and re-emergence of major transboundary and emerging animal diseases have raised serious global concerns, both financially and in terms of public health. These diseases directly affect food safety by limiting the availability and quality of high-value animal products [1]. Among these, lumpy skin disease (LSD), a re-emerging viral infection affecting cattle (*Bos taurus and Bos indicus*) and water buffaloes (*Bubalus bubalis*), poses significant economic challenges. Owing to its transboundary nature and growing impact, it is classified as a notifiable disease by the World Organization for Animal Health (OIE) [2, 3]. The disease has been referred to by various names, including “Neethling virus disease,” “exanthema nodularis bovis,” “pseudo-urticaria,” and “knopvelsiekte.” However, among these terms, “lumpy skin disease” (LSD) remains the most widely used designation [4, 5]. LSD is caused by LSD virus (LSDV), which belongs to the genus *Capripoxvirus* within the family Poxviridae. Along with cattle and buffalo, the virus can also infect different wild animals, including giraffes, impalas, wildebeest, springboks, and oryxes [6, 7]. However, goats and sheep are resistant to infection, even when they are kept close to infected cattle and buffalo [8].

LSD is a vector-borne disease, and the transmission of LSD occurs through various blood-sucking arthropods. These include mosquitoes, such as *Culex mirificens* and *Aedes natrionus*; biting flies, such as *Stomoxys calcitrans* and *Biomyia fasciata*; and male ticks, including *Rhipicephalus appendiculatus* and *Amblyomma hebraeum* [7]. LSD typically results in high morbidity (~70%–90%), with low to moderate mortality (~7%–10%) [3, 9]. The severity can range from mild, subclinical cases to fatal outcomes, depending on factors such as the virus strain, vector abundance, and age and immune status of the affected animal [2, 5]. LSD is characterized by a range of clinical signs, including high fever, nasal discharge, excessive tearing (epiphora), and progressive emaciation. Affected animals may also exhibit swollen lymph nodes (lymphadenitis) and the development of papules and nodules measuring 0.5–5.0 cm in diameter. These skin lesions commonly appear on various parts of the body, including the head, neck, udder, scrotum, perineum, and mucous membranes of the mouth, respiratory tract, and genitalia [7, 10]. LSD causes economic losses through reduced productivity, animal deaths, and increased costs for diagnosis, treatment, and trade restrictions.

Since its emergence in Zambia, Africa, in 1929, LSD has spread globally for more than 90 years, becoming endemic across Africa and rapidly reaching previously disease-free countries [11, 12]. LSD was first reported in the Middle East in 1988 in Egypt and in 2005 in Bahrain, remaining confined to the region (western Asia) until 2018 [13, 14]. LSD was first reported in South Asia in 2019 and affects China, Bangladesh, India, and Nepal [14, 15]. India's first outbreak occurred in Odisha before it spread nationwide [16]. By 2020, the disease had spread to Nepal, Sri Lanka, Bhutan, Bangladesh, Vietnam, and Southeast China [14, 17, 18]. Bangladesh emerged as the first hotspot, with the earliest case recorded on July 14, 2019 [19].

Disease occurrence follows temporal patterns that can be categorized as short-term, cyclical, seasonal, or long-term, with time series analysis commonly used to study these trends. A seasonal trend is a specific type of cyclical pattern where disease cases rise and fall at regular intervals due to seasonal changes [20]. Seasonal variation, often driven by changes in vector populations, is particularly important in understanding the dynamics of vector-borne diseases and developing effective control measures [21]. Epidemics of LSD occur during the rainy season, when arthropod vector populations are abundant, whereas LSD incidence sharply decreases during the dry and cold weather seasons [22]. The resurgence of the disease has been consistently associated with climate factors, including high rainfall, humidity, temperature, and wind speed, which affect vector populations and activity [23].

Monitoring animal disease data is essential for understanding a country's disease status. Analyzing spatial and temporal patterns is crucial for planning effective surveillance and control strategies. Despite the large number of LSDs reported in Bangladesh, there have been no studies on the spatiotemporal analysis of LSD and the effects of climate factors on the incidence of LSD. Therefore, this study aimed to assess the spatiotemporal distribution of LSD incidence in Bangladesh and to determine the correlation between LSD incidence and climate factors using data from 2020 to 2023.

## 2. Materials and Methods

### 2.1. Study Area and Data

Bangladesh is located in South Asia, bordering India and Myanmar. The regional states and city administrations in Bangladesh are divided into 491 subdistricts (also known as Upazila), 64 districts, and eight divisions. The Bangladesh livestock population is estimated to consist of 23.785 million cattle and 1.471 million buffalo [24]. As part of disease surveillance, cases (single cattle or buffalo) that are treated at Upazila veterinary hospitals are regularly documented. The Department of Livestock Services (DLS) Epidemiology Cell receives monthly surveillance data from subdistricts, which are then forwarded to districts and divisions. For this investigation, LSD cases in cattle and buffalo from 2020 to 2023 were sourced from DLS (https://dls.gov.bd/) and aggregated at the district level, which is the administrative unit used for reporting livestock diseases in Bangladesh. The DLS maintains records of LSD cases through routine passive surveillance, whereby field veterinarians report clinically diagnosed cases based on animal examinations and history. For this study, we included only data where the animals presented all of the clinical symptoms of LSD, such as several clinical symptoms, including skin nodules, swollen lymph nodes, fever, nasal discharge, lacrimation, and mucous membrane and internal organ edema [25, 26]. In Bangladesh, LSD is one of the major illnesses affecting cattle and buffalo that can present with a combination of characteristic symptoms, making it clinically distinguishable from many other diseases, although differential diagnoses should also be considered.

### 2.2. Meteorological Data

Meteorological data for this study were obtained from the Bangladesh Meteorological Department (BMD) (https://live7.bmd.gov.bd/). The dataset included station-based measurements of rainfall, relative humidity, maximum temperature, minimum temperature, average sunshine, and average temperature, all of which were provided in comma-separated value (CSV) format. To facilitate the analysis, the station-based data were aggregated to represent overall district-level and nationwide averages for Bangladesh. Using the “tidyverse*”* package (R programing, version 4.4.2), the data were transformed from a wider format to a longer, tidy format to streamline data manipulation and facilitate statistical modeling.

### 2.3. Statistical Analysis

A general descriptive summary and box-and-whisker plots were used to analyze the spread time and determine the LSD case distribution in Bangladesh. A box plot provides a visual representation of the distribution. The box extends from the bottom hinge, which is the 25th percentile, to the higher hinge, which is the 75th percentile. The line across the box represents the median.

The identification of geographical clusters can be simplified by hotspot analysis. To identify the statistically significant high values (hot spots) and low values (cold spots), the study utilizes the Getis-Ord Gi^*∗*^ statistics (Gi^*∗*^. According to the Gi^*∗*^ [27, 28], hotspots are spatial clusters with high correlations that are located within a given radius of the entire study region. If there is no discernible global spatial clustering, hotspot detection is sensitive when local spatial events are clustered [29]. When both the disease values and those of the surrounding areas are relatively high, the location is identified as a hot zone. This is assessed using the Gi^*∗*^, which calculates a *Z*-score for each feature by comparing the local sum of values within a specified neighborhood to the expected sum under spatial randomness. A high positive *Z*-score indicates a significant clustering of high values (hot spot), while a low negative *Z*-score reflects a clustering of low values (cold spot). The statistical significance of these clusters is determined by the magnitude of the *Z*-scores, with thresholds of ±1.65, ±1.96, and ±2.58 corresponding approximately to 90%, 95%, and 99% confidence levels, respectively. Thus, the greater the absolute value of the *Z*-score, the stronger the evidence for spatial clustering, with the associated *p*-values confirming the reliability of the detected hot and cold spot patterns. Here, the Gi^*∗*^ [27] is calculated. All contiguity matrices used in this analysis were constructed via R version 4.4.2 and the spdep and *ggplot2* packages [30, 31].

To determine the features of the global pattern (clustered, dispersed, and random) of LSD and to determine whether significant spatial autocorrelation was present, the global Moran's *I* statistic was employed as a spatial correlation measure [28, 32]. There is a positive correlation in the spatial distribution when the Moran's *I* index is greater than zero, and a negative correlation in the spatial distribution when the Moran's *I* index is less than zero. The Moran's *I* index ranges from +1 to −1. A positive association is more evident when the Moran's *I* index value is greater. A random geographical distribution is indicated when Moran's *I* index = 0 [33]. The formula for Moran's *I* is based on Mandal et al. [34]. Global Moran's *I* was used to determine whether spatial autocorrelation existed across the whole study region rather than just at particular points in time. The risks of LSD were then evaluated via univariate and bivariate cluster analyses. Local indicators of spatial association (LISA) and univariate global Moran's *I* are examples of univariate studies. To find local clusters and evaluate their significance, LISA, the decomposition of univariate global Moran's *I* into individual regions, was used [35]. As an extension of univariate cluster analyses [36], bivariate cluster analysis, which includes bivariate global Moran's *I* and bivariate LISA, was conducted to determine the spatial correlation between two variables. This analysis was performed with *spdep*, and visualized the maps with the *ggplot2* package in R programing [30, 31].

To investigate the associations between the number of LSD cases and meteorological parameters, Spearman's rank correlation was first employed. This nonparametric method was used to identify potential relationships between LSD cases and weather variables, including rainfall, relative humidity, maximum temperature, minimum temperature, average temperature, and average sunshine. This preliminary analysis facilitated the selection of relevant weather variables as external repressors for further modeling. Multivariate regression techniques, including Poisson regression, quasi-Poisson regression, and negative binomial regression, were subsequently conducted to quantify and model the relationships between the LSD cases and the selected meteorological variables. These regression methods account for overdispersion and other potential statistical complexities in the count data, ensuring robust analysis. The dependent variable for the multivariate regression analyses (Poisson, quasi-Poisson, and negative binomial regression) was the number of reported LSD cases. The independent variables, included in all models, comprised key meteorological factors: rainfall (mm), relative humidity (%), maximum temperature (°C), minimum temperature (°C), average temperature (°C), average sunshine duration (h), and wind speed (m/s). Prior to model fitting, we assessed multicollinearity among the explanatory variables using the variance inflation factor (VIF) (Table S1). Multicollinearity was detected among some of the explanatory variables, particularly the temperature indicators (average, minimum, and maximum temperature), as reflected by high VIFs. While multicollinearity can inflate standard errors and reduce the precision of coefficient estimates, it does not bias the estimates or invalidate the overall model performance in generalized linear models (GLMs) such as Poisson, quasi-Poisson, and negative binomial regression [37–43]. Therefore, instead of omitting theoretically important variables, which could lead to model misspecification and loss of critical climatic information, all predictors were retained. Furthermore, all models were implemented without incorporating time-lagged features, focusing instead on the contemporaneous effects of climatic conditions on LSD cases. While lagged effects between climate variables and disease outcomes are often important to consider, evidence suggests that incorporating lagged terms can become methodologically burdensome and may yield limited gains; particularly when sample sizes are modest or the temporal delay is uncertain. Adding lag variables may lead to greater loss of model precision with minimal improvement in capturing lag effects, thus justifying the preference for simpler, immediate-effect models when appropriate [44, 45]. The correlation was performed with the “*corrplot”* package, and the regressions “*glm”* and “*MASS”* packages were used [46–48].

## 3. Results

### 3.1. Descriptive and Temporal Patterns

The mean number of LSD cases reported was 3971 (standard deviation [SD] = ±5132), indicating high variability. The average rainfall was 186 mm (SD = ±174 mm), whereas the mean relative humidity was 79.3% (SD = ±5.1%). The maximum temperature averages 34.68°C (SD = ±2.82°C), whereas the minimum temperature has a mean of 18.1°C (SD = ±5.7°C). The average sunshine duration is 5.60 h (SD = ±1.26 h), and the overall average temperature is 25.6°C (SD = ±4.1°C). The mean wind speed was 1.37 m/s (SD = ±0.45 m/s), reflecting moderate variation (Table 1).

**Figure 1:**
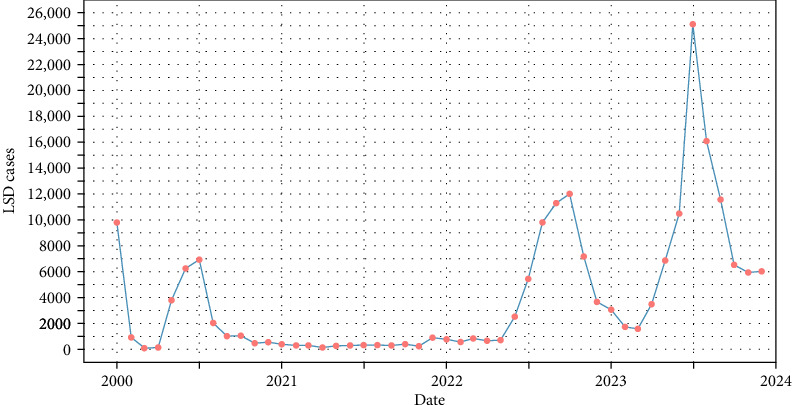
Yearly trend plot of LSD cases from 2020 to 2023.

The yearly trend plot (Figure 1) of LSD cases from 2020-2023 shows a cyclical pattern with periodic outbreaks. A sharp spike occurred in early 2020, followed by a decline and a subsequent increase. The number of cases remained low throughout 2021 but began increasing again in late 2022. A major outbreak peaked in 2023, reaching its highest point before declining and showing another resurgence. By late 2023, cases had dropped but remained present.

**Figure 2:**
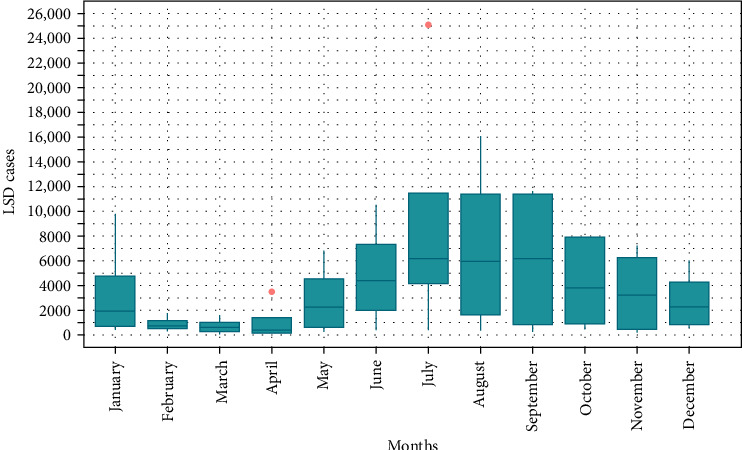
Monthly boxplot distribution of LSD cases from 2020 to 2023.

The monthly distribution of LSD cases (Figure 2) highlights seasonal trends. The number of cases remained relatively low from February to April, with a gradual increase starting in May. A significant surge is observed from June, peaking between July and September, indicating the highest median and variability during these months. After September, cases begin to decline but remain relatively high through October before dropping further in November and December. The presence of outliers in January, April, and July suggests occasional extreme case surges.

### 3.2. Spatiotemporal Pattern

**Figure 3:**
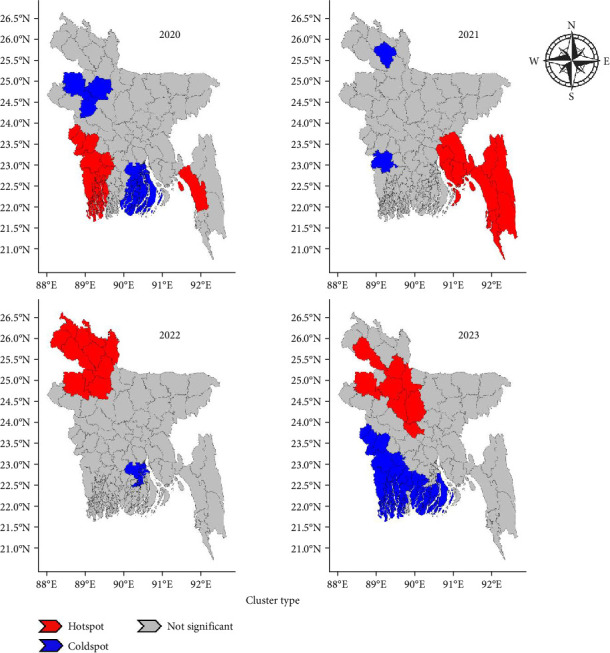
LSD hotspot development from 2020 to 2023. The red color represents hotspots (high LSD outbreaks clustered), the blue color represents cold spots (low LSD outbreaks clustered), and the gray color indicates that this dot was not significant.

From 2020 to 2023, the Gi^*∗*^ maps for LSD outbreaks in Bangladesh revealed distinct spatial patterns over time (Figure 3). In 2020, a significant hotspot (red) concentrated in the southwestern and southeastern regions, whereas a coldspot (blue) appeared in the northern and southern coastal areas. By 2021, the southeastern region remained a hotspot, and blue cold spots had become reduced, except for a small cluster in the north. In 2022, the hotspot shifted toward the northern and northwestern regions, whereas a cold spot emerged in the southern region. By 2023, the northern region continues to exhibit hotspot central regions that remain largely nonsignificant throughout the study period, indicating minimal clustering.

These shifts highlight dynamic epidemiological trends, with hotspots transitioning from southern to northern Bangladesh over time, whereas southern Bangladesh gradually became a cold spot, suggesting evolving epidemiological trends and spatial dynamics.

**Figure 4:**
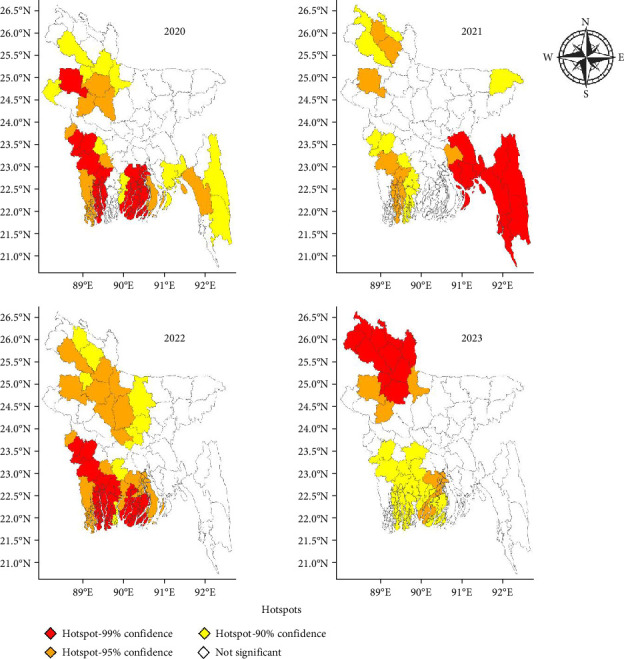
LSD hotspot development in Bangladesh from 2020 to 2023 on the basis of Gi Bin^*∗*^ analysis. Red areas represent hotspots (high LSD outbreak clustering) with 99% confidence, and orange and yellow areas indicate hotspots with 95% and 90% confidence, respectively.

The Getis-Ord Gi Bin^*∗*^ (Gi Bin^*∗*^) maps from 2020 to 2023 reveal notable spatial and temporal variations in LSD outbreaks across Bangladesh, highlighting regions with significant clustering patterns (Figure 4). In 2020, the southeastern and southeastern regions emerged as primary hotspots with 99% confidence, whereas lower confidence clusters (95% and 90%) appeared in the northern and central parts. By 2021, the southeastern region remains the dominant hotspot with 99% confidence, whereas the northern and central regions show scattered lower-confidence clusters. A major shift occurred in 2022, as new hotspots emerged in the northwest, whereas the southwestern region continues to exhibit high clustering. In 2023, the hotspot pattern shifts further north, with the northwestern region now showing the most significant clustering at the 99% confidence level. Moreover, the southwestern region exhibited a decline in hotspot intensity, with lower confidence clusters persisting in the central and southern parts. These patterns indicate a dynamic shift in LSD outbreak intensity, with the southeastern region initially being the dominant hotspot, but later seeing hotspots emerge in the northwest. The results highlight evolving epidemiological trends, emphasizing the need for region-specific disease control strategies.

**Figure 5:**
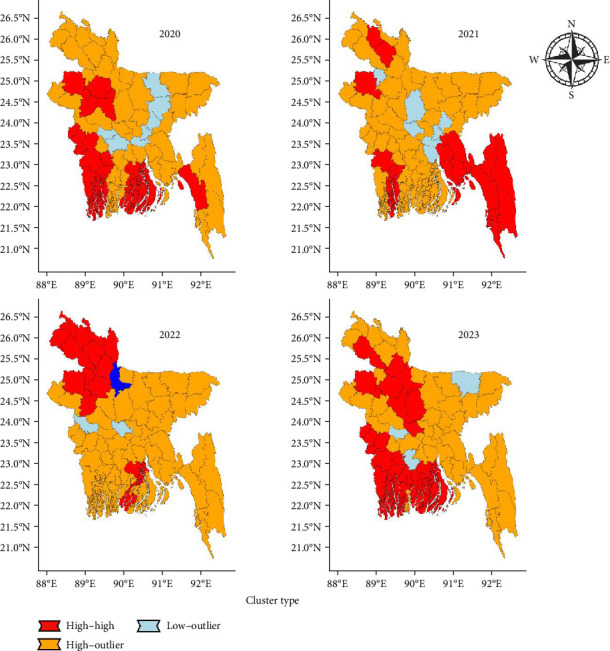
LISA (local indicators of spatial association) analysis for LSD outbreaks in Bangladesh from 2020 to 2023. Red regions indicate “high–high” clusters (significant hotspots), orange regions represent “high–low” or “low–high” outliers, and blue regions signify “low–low” clusters (coldspots).

After conducting LISA analysis, maps representing the spatial clustering of LSD outbreaks were prepared (Figure 5). Between 2020 and 2021, high–high hotspots were consistently observed in southeastern coastal regions, particularly in Chattogram and Cox's Bazar, indicating that these areas are major epicenters of LSD outbreaks. Additionally, in 2020, clusters were prominent in southwestern districts, such as Khulna, while low-outlier regions were scattered in the north.

In 2021, the southeastern region remained a significant hotspot, whereas northern districts, including parts of Rangpur and Dinajpur, started showing high-outlier patterns, indicating a shift from previous years. By 2022, the northern regions became dominant high–high clusters, whereas low outliers were present in some central regions, indicating potential reductions in LSD activity there. The southwestern coastal regions maintained clustering patterns, but their intensity appeared to decrease compared with that in earlier years. By 2023, high–high hotspots continued to be concentrated in the northern district, particularly in Rajshahi and Rangpur, while the southeastern regions, although still active, showed a slightly reduced intensity. Low outliers persisted in some southern areas, suggesting localized declines in outbreaks. High outliers remained dispersed across central districts, reflecting fluctuating LSD activity.

Across the years, the southeastern region remained a persistent hotspot, whereas the northern region transitioned from a low-outlier area in the early years to a significant high–high cluster. The shifting clustering patterns indicate an evolving epidemiological landscape, emphasizing the need for targeted disease control interventions in both persistent and emerging hotspot areas.

### 3.3. Correlation and Regression Analysis

**Figure 6:**
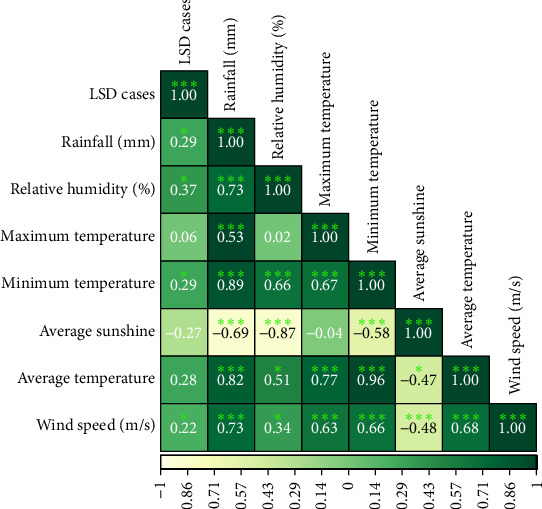
Spearman's rank correlation between LSD cases and meteorological factors.

Spearman rank correlation analysis revealed that LSD cases were significantly positively associated with rainfall (*r* = 0.29, *p* < 0.05), relative humidity (*r* = 0.37, *p* < 0.05), minimum temperature (*r* = 0.29, *p* < 0.05), and wind speed (*r* = 0.22, *p* < 0.05), indicating that higher humidity, increased precipitation, warmer minimum temperatures, and stronger wind speed may contribute to LSD outbreaks (Figure 6).

In the Poisson model, all the meteorological variables significantly influenced the LSD cases. Rainfall showed a 0.5% increase per mm (incidence rate ratio [IRR] = 1.005, *p*  < 0.001), relative humidity a 19.3% increase per 1% rise (IRR = 1.193, *p*  < 0.001), maximum temperature a 46.6% increase per 1°C rise (IRR = 1.466, *p*  < 0.001), and minimum temperature a 68.7% increase per 1°C rise (IRR = 1.687, *p*  < 0.001). The average sunshine had the greatest impact, increasing LSD cases by 257.5% per hour of increase (IRR = 3.575, *p*  < 0.001), whereas the wind speed showed a 163.9% increase per m/s (IRR = 2.639, *p*  < 0.001). Conversely, the average temperature was associated with a 63.8% decrease per 1°C increase (IRR = 0.362, *p*  < 0.001), indicating a strong protective effect (Table 2). In the quasi-Poisson model, the significance of some variables change due to overdispersion correction. Minimum temperature remained a significant predictor, increasing LSD cases by 68.7% per 1°C increase (IRR = 1.687, *p*=0.022), whereas average sunshine increased cases by 257.5% per hour increase (IRR = 3.575, *p*=0.001). The average temperature still had a strong protective effect, reducing LSD cases by 63.8% per 1°C increase (IRR = 0.362, *p*=0.010). The wind speed also remained significant, leading to a 163.9% increase per m/s rise (IRR = 2.639, *p*=0.043). Rainfall, relative humidity, and maximum temperature were no longer statistically significant (Table 2). In the negative binomial model, rainfall showed a 0.6% increase per mm (IRR = 1.006, *p*=0.033), the minimum temperature increased LSD cases by 80.5% per 1°C rise (IRR = 1.805, *p*=0.004), and average sunshine increased cases by 227.6% per hour rise (IRR = 3.276, *p*  < 0.001). The average temperature remained a strong protective factor, reducing LSD cases by 68.9% per 1°C increase (IRR = 0.311, *p*=0.001). The relative humidity, maximum temperature, and wind speed were no longer statistically significant in this model (Table 2).

## 4. Discussion

In Bangladesh, the monsoon (rainy) season typically starts from June to October, while the winter season is from November to February, and most LSD cases were found in this month in our study. LSD is more common in the wet summer and autumn months, especially in low-lying regions and areas near watercourses [49]. The highest seasonal factor is observed in October, whereas the lowest occurs in May [50]. In Middle East countries, the highest number of LSD outbreaks was reported between September and February [51]. EFSA et al. [16] reported mostly outbreaks from June to October in Turkey.

From 2020 to 2021, the LSD hotspots were located mainly in the southwest and southeast regions. LSD outbreaks in the southwest and southeast regions may be linked to factors such as cross-border cattle trade, increased livestock movement (especially during Eid-ul-Adha), and breeding practices, including local zebu and crossbreds such as Holstein Friesian or Sahiwal. Southeast Bangladesh, which serves as a major trade hub, reported the first cases, whereas Southwest Bangladesh also experienced significant infections, likely due to high levels of cattle movement and weakened immunity from stress factors such as pregnancy [52–54]. The hotspot gradually shifted to the northwest regions of Bangladesh. This shift may be due to these areas sharing borders with India, where illegal cattle movement occurs. This is also supported by molecular studies demonstrating that LSDV strains circulating in Bangladesh and India reveal near-identical GPCR (99.7%–100%), RPO30 (100%), and EEV (100%) gene sequences, strongly suggesting a common exotic source of introduction and reinforcing the likelihood of cross-border transmission [55]. However, outbreaks have occurred mostly within 4–5 km of infected farms; thus, after the introduction of newly affected cattle, this disease spreads rapidly. Additionally, local farmers often share grazed lands, which makes it easier for the disease to spread [56–59]. Another study revealed that spatial cluster analysis identified hotspots in the Aegean, Southeastern, and Eastern regions, revealing a positive spatial autocorrelation of LSD cases across Turkey [60].

Hotspot analysis (Gi^*∗*^) of the deviance residuals identified the Shamli, Muzaffarnagar, and Gautam Buddha Nagar districts in Uttar Pradesh, India, as significant hotspots [61]. Swiswa et al. [62] reported that between 2005 and 2009, significant disease hotspots persisted in the southern, northern, and western regions of the country, whereas new hotspots emerged in central areas, particularly in large-scale commercial farmlands. LISA analysis highlights the spatial diversity in LSD outbreak distributions across the region. Most districts displayed clustering patterns of high–high or low–low occurrences, indicating localized variations on the basis of case numbers. Notably, outliers with low–high and high–low spatial autocorrelation patterns added complexity to the spatial dynamics. Consistent with the choropleth map findings, areas with high LSD cases clustered together were primarily concentrated in the eastern and northern regions of Thailand [63].

In 2022 and 2023, high–high clusters were mainly concentrated in the eastern and central regions of Java, with fewer occurrences in the western part. In 2022, low–low clusters were predominantly found in western Java, with some scattered in the central and eastern regions. However, by 2023, the number of low–low clusters had significantly declined, indicating a notable shift in the spatial distribution pattern of LSD outbreaks [64]. A space-time cluster was detected, which lasted for 3 years from January 1, 2002, to December 31, 2005. This cluster covered 24 districts in the Eastern and Central Regions and two districts in the Northern Region. During this period, 383 outbreaks were recorded, which was significantly greater than the expected 137.97 outbreaks in Uganda [65]. The introduction of new animals, water supply sources, and farm floor types (brick or cemented) are potential risk factors for LSD outbreaks, which are major causes of highly clustered areas [15]. LSD clusters are influenced by the introduction of new animals, grazing in vector-prone areas, poor biosecurity measures, and inadequate vector control, especially during the summer and rainy seasons [66].

In the Spearman rank correlation, rainfall, humidity, minimum temperature, and wind speed were significantly correlated. Thus, a correlation between the average precipitation rate and LSD outbreaks was identified [67, 50]. Temperature and precipitation were found to be significantly correlated with LSDV [51]. The LSD risk was positively associated with both precipitation and temperature [68]. Abdelgawad [69] reported significant correlations between LSD outbreaks and temperature, wind speed, and humidity.

Our study revealed that, in the Poisson model, rainfall (mm), relative humidity (%), maximum temperature, minimum temperature, average sunshine, and wind speed (m/s) significantly increase LSD incidence, whereas in the quasi-Poisson model and negative binomial model, minimum temperature, average sunshine, and wind speed (m/s) significantly increase LSD incidence. Studies have shown that higher temperatures or the summer season are the most vulnerable to LSD transmission, and that there is a significant association between increasing LSD transmission and these conditions [68, 70]. Agrawal et al. [61] reported that temperature and humidity are significantly associated with increased LSDV in the eastern district of Uttar Pradesh, India [69]. reported that temperature, wind speed, and humidity affect the number of LSD outbreaks. This could be because mosquitoes and other vectors tend to be more active during the summer season [68, 70–74]. In addition, humid conditions create a favorable environment for the breeding of biting flies, which are known to transmit LSD [65]. Outbreaks of LSD have also been reported after periods of heavy rainfall or monsoon conditions, likely due to an increase in vector activity [71, 73–76]. Strong wind patterns may also facilitate the long-distance spread of LSDV-infected vectors, such as stable flies, which are more robust fliers than mosquitoes are [77, 78]. A study analyzing the potential atmospheric long-distance dispersal of LSDV from Egypt to Israel revealed that wind trajectories established connections between outbreak locations, suggesting that wind facilitates transmission [77]. After the rainy season (October to December), a higher incidence of LSD outbreaks was reported by Tesfaye et al. [79]. Negative binomial regression analysis suggested that the IRR of LSD is expected to increase with increasing temperature and relative humidity [80].

Considering the substantial impact of climatic factors on the transmission patterns of LSD, the implementation of comprehensive preventive strategies is crucial to mitigate its burden in Bangladesh. Vaccination remains the cornerstone of LSD control, with homologous live attenuated vaccines being widely used to induce herd immunity and reduce the severity of outbreaks. In addition, heterologous vaccines, such as sheep- and goat-pox vaccines, have been utilized in certain regions owing to their demonstrated cross-protective efficacy against LSDV. The government has approved the use of both locally developed and imported live-attenuated vaccines, including the homologous Neethling strain and goat pox–based heterologous vaccines that provide some cross-protection against LSDV [81]. Complementary preventive measures, including the enforcement of strict biosecurity protocols, quarantine of infected or exposed herds, regulation of animal movement, targeted vector control interventions (particularly against mosquitoes, flies, and ticks), and the adoption of improved husbandry practices by increasing farmer awareness through outreach programs, play a critical role in limiting disease transmission [73, 81–83].

The spread of LSD in cattle is influenced by multiple factors, including climatic conditions, livestock movement, and vaccination strategies. While various modeling approaches exist, such as spatial diffusion models that incorporate animal movement and vaccination [84], geographically weighted regression (GWR) models to assess environmental effects [61], kernel-based transmission models to estimate spatial spread probabilities [85], deep learning models for early detection from cattle images [86], and spatiotemporal models analyzing outbreak clusters [87]. Our study was constrained by the availability of only area-level LSD case reports, without detailed information on livestock movements, herd-level vaccination coverage, or vector dynamics. Given these data limitations, we applied spatial statistical methods, including Gi^*∗*^ to detect local hot and cold spots, global Moran's *I* and LISA to quantify spatial autocorrelation, bivariate LISA to examine environmental associations, and Poisson, quasi-Poisson, and negative binomial regression to evaluate relationships with climatic factors. These methods are well-established for analyzing spatial patterns when detailed mechanistic data are unavailable, providing robust insights into disease clustering and environmental drivers. Other modeling approaches, such as spatial diffusion models, kernel-based transmission models, or deep learning-based detection models, could not be applied in this study because the necessary data; such as livestock movement, vaccination strategies, cattle images, land use types (fallow, pasture, nonagricultural land), predicted LSD incidence, cattle grazing distances, or real-time satellite measurements were not available.

This study examined the associations between meteorological factors (temperature, humidity, and precipitation) and LSD. However, several other important factors that may influence LSD transmission were not included in the analysis. These include herd size, grazing practices, housing conditions, feeding management, vector control measures, livestock movement patterns, vaccination status, biosecurity practices, genetic susceptibility of cattle, and overall farm management strategies. The exclusion of these factors may limit the comprehensiveness of our findings. Future research should incorporate these variables to provide a more holistic understanding of LSD risk factors and improve disease prevention and control strategies.

Our findings provide important insights that can be utilized to strengthen the control of LSD in Bangladesh. First, the results can be applied to develop robust forecasting tools, thereby improving the prediction of outbreak trends across different regions and seasons. Second, the epidemiological associations demonstrated in this study can support in evaluating the potential impact of intervention strategies such as vaccination, targeted culling, or movement restrictions, by simulating how these measures may influence outbreak patterns under different climatic conditions. Third, the identification of high-risk periods provides evidence to guide the timing and prioritization of vaccination campaigns. The strategic implementation of vaccination programs before peak vector activity can enhance herd immunity, reduce transmission pressure, and ultimately minimize the economic and animal health impacts of LSD.

## 5. Conclusion

This study highlights the significant temporal and spatial variability of LSD outbreaks in Bangladesh from 2020 to 2023, with clear seasonal patterns and evolving hotspot regions. These findings underscore that LSD outbreaks peak during the monsoon months (July–September) and are strongly influenced by meteorological variables such as rainfall, humidity, sunshine duration, and temperature. Spatial analyses revealed a dynamic shift in outbreak hotspots from the southeastern coastal areas to the northern and northwestern districts over time, indicating an evolving epidemiological landscape. The statistically significant correlations and regression outcomes confirmed the critical role of environmental factors in modulating the incidence of LSD. To mitigate LSD outbreaks, surveillance and early warning systems should be strengthened during the peak season (May–September), and weather data should be used to improve predictive models. Control efforts must focus on emerging hotspots in northern Bangladesh and northwestern Bangladesh while maintaining vigilance in historically affected southeastern Bangladesh. Enhancing veterinary services, vaccination coverage, and timely reporting is essential. Farmer education and community engagement should promote awareness and early action. Continued research is needed to better understand environmental influences and improve forecasting tools.

## Figures and Tables

**Table 1:** Descriptive statistics of the LSD cases and the meteorological factors from 2020 to 2023.

Characteristics	Minimum	Maximum	Mean (SD)
LSD cases	86	25,084	3971 (5132)
Rainfall (mm)	0	534	186 (174)
Relative humidity (%)	68.8	86.3	79.3 (5.1)
Max temperature (°C)	28.30	39.29	34.68 (2.82)
Minimum temperature (°C)	8.3	25.1	18.1 (5.7)
Average sunshine (h)	3.16	7.81	5.60 (1.26)
Average temperature (°C)	17.4	29.7	25.6 (4.1)
Wind speed (m/s)	0.85	2.29	1.37 (0.45)

Abbreviations: m/s, meter/second; mm, millimeter; SD, standard deviation.

**Table 2:** Poisson, quasi-Poisson, and negative binomial models are used to measure the effects of meteorological factors on LSD.

Variables	Poisson model			Quasi-Poisson model			Negative binomial model		
	IRR	95% CI	*p*-Value	IRR	95% CI	*p*-Value	IRR	95% CI	*p*-Value
Rainfall (mm)	1.005	1.005, 1.005	**0.000**	1.005	0.999, 1.011	0.089	1.006	0.999, 1.014	**0.033**
Relative humidity (%)	1.193	1.189, 1.198	**0.000**	1.193	0.983, 1.478	0.096	1.120	0.932, 1.338	0.177
Max temperature	1.466	1.453, 1.479	**0.000**	1.466	0.872, 2.483	0.157	1.531	0.944, 2.566	0.108
Minimum temperature	1.687	1.675, 1.700	**0.000**	1.687	1.109, 2.630	**0.022**	1.805	1.132, 2.829	**0.004**
Average sunshine	3.575	3.532, 3.619	**0.000**	3.575	1.802, 7.394	**0.001**	3.276	1.678, 6.374	**0.000**
Average temperature	0.362	0.357, 0.366	**0.000**	0.362	0.168, 0.731	**0.010**	0.311	0.147, 0.667	**0.001**
Wind speed (m/s)	2.639	2.598, 2.680	**0.000**	2.639	1.064, 6.608	**0.043**	2.515	0.965, 6.983	0.058

*Note:* Significant values are in bold (*p* < 0.05).

Abbreviations: CI, confidence interval; IRR, incidence rate ratio.

## Data Availability

Upon reasonable request, the corresponding author can provide access to the datasets used and/or analyzed in this study.

## Nomenclature

LSD: Lumpy skin disease
DLS: Department of Livestock Services
BMD: Bangladesh Meteorological Department
CSVs: Comma-separated values
LISA: Local indicator of spatial association
SD: Standard deviation
IRR: Incidence rate ratio
CI: Confidence interval
LSDV: Lumpy skin disease virus.

## Supporting Information

Supporting Information Table S1. Variance inflation factor.
