# Supplementary material for: Spatiotemporal Distribution and Meteorological Determinants of Lumpy Skin Disease (LSD) Occurrence in Bangladesh From 2020 to 2023
Source: Transbound Emerg Dis. 2025 Nov 14;2025:4259023. doi: 10.1155/tbed/4259023 (PMC12638142; doi:10.1155/tbed/4259023)
Supplement: Supporting Information — Table S1. Variance inflation factor. [file 4259023.f1.docx]

**Supplementary Table 1: Variance inflation factor**

| **Factors** | **VIF** | **1/VIF** |
| --- | --- | --- |
| Average Temperature | 83.478 | 0.012 |
| Minimum Temperature | 44.28 | 0.023 |
| Maximum Temperature | 22.014 | 0.045 |
| Rainfall | 4.683 | 0.214 |
| Average sunshine | 3.59 | 0.279 |
| Wind speed | 2.055 | 0.487 |
| RH | 1.898 | 0.527 |
| Mean VIF | 23.143 | 0.043 |
